# Multimorbidity patterns and blood biomarkers of Alzheimer's disease in community‐dwelling cognitively unimpaired older adults

**DOI:** 10.1002/alz.70411

**Published:** 2025-06-22

**Authors:** Alessandra Marengoni, Giulia Grande, Martina Valletta, Caterina Gregorio, Amaia Calderón‐Larrañaga, Matilda Dale, Claudia Fredolini, Bengt Winblad, Davide Liborio Vetrano

**Affiliations:** ^1^ Aging Research Center, Department of Neurobiology, Care Sciences and Society Karolinska Institutet and Stockholm University Solna Sweden; ^2^ Department of Clinical and Experimental Sciences University of Brescia Brescia Italy; ^3^ Stockholm Gerontology Research Center Stockholm Sweden; ^4^ Affinity Proteomics Stockholm, Science for Life Laboratory, Department of Protein Science, School of Engineering Sciences in Chemistry, Biotechnology and Health (CBH) Royal Institute of Technology (KTH) Solna Sweden; ^5^ Division of Neurogeriatrics, Department of Neurobiology, Care Sciences and Society Karolinska Institutet Solna Sweden; ^6^ Theme Inflammation and Aging Karolinska University Hospital Huddinge Sweden

**Keywords:** Alzheimer's disease, blood biomarkers, community, multimorbidity, patterns

## Abstract

**INTRODUCTION:**

Alzheimer's disease (AD) blood biomarkers hold clinical potential but their concentration may vary with somatic conditions.

**METHODS:**

We investigated the concentration of six AD blood biomarkers in relation to multimorbidity as disease count and four multimorbidity patterns in 2290 cognitively unimpaired older adults.

**RESULTS:**

Levels of phosphorylated tau (p‐tau)181, p‐tau217, neurofilament light chain (NfL), and glial fibrillary acidic protein (GFAP) increased with increasing number of diseases. In multi‐adjusted regressions, compared to individuals without multimorbidity, the anemia/sensory impairment pattern was associated with altered levels of all biomarkers except amyloid beta (Aβ)42/40, GFAP, and total tau (p‐tau181: β = 0.18, 95% confidence interval [CI]: 0.08, 0.28; p‐tau217: β = 0.11, 95% CI: 0.03, 0.18; NfL: β = 0.14, 95% CI: 0.06, 0.21) and the cardiometabolic/inflammatory pattern was associated with altered levels of all biomarkers except Aβ42/40 and GFAP (p‐tau181: β = 0.24, 95% CI: 0.12, 0.36; p‐tau217: β = 0.23, 95% CI: 0.14, 0.32; NfL: β = 0.32, 95% CI: 0.23, 0.40; total tau: β = 0.23, 95% CI: 0.07, 0.39). Results remained unchanged after excluding those who developed dementia in 15 years.

**DISCUSSION:**

More diseases and specific multimorbidity patterns altered the levels of several AD blood biomarkers, highlighting caution when using them in adults with complex health profiles.

**Highlights:**

- In cognitively unimpaired older adults blood biomarkers of Alzheimer's disease varied depending on the number of chronic diseases and specific patterns of multimorbidity.
- Phosphorylated tau (p‐tau)181, p‐tau217, neurofilament light chain (NfL), and glial fibrillary acidic protein levels increased along with increasing numbers of chronic diseases.
- P‐tau181, p‐tau217, and NfL levels were significantly higher in individuals in the anemia/sensory impairment and cardiometabolic/inflammatory multimorbidity patterns compared to those without multimorbidity.
- Results remained unchanged after excluding participants who developed dementia during 15‐year follow‐up.

## 1 BACKGROUND

Blood biomarkers of Alzheimer's disease (AD) have emerged as reliable indicators of AD pathology and neurodegeneration^1^ and have been shown to accurately predict future cognitive decline, including the onset of dementia.^2^, ^3^ Their scalability and non‐invasiveness make them promising candidates for integration into the clinic, including primary care settings.^4^, ^5^, ^6^, ^7^

However, despite their potential, several factors must be considered before their clinical implementation. Blood concentrations of these biomarkers may vary due to conditions that either reflect a higher AD pathology burden or alter the metabolism and bioavailability of these proteins. Such alterations can lead to false positive results and misclassification. Studies have shown that a higher number of co‐occurring chronic diseases, as well as elevated Charlson Comorbidity Index scores, are linked to variations in blood biomarker levels.^8^, ^9^ Additionally, specific conditions like chronic kidney disease, obesity, diabetes, hypertension, and cancer have been shown to impact blood biomarker concentration.^9^, ^10^, ^11^

Along with the potential of AD blood biomarkers to be used in the clinic, identifying the factors that influence their levels becomes essential to accurately interpret results and establish reference ranges.^12^ This is especially important in community settings as well as in primary care, where populations are more heterogeneous than in memory clinics or other specialist settings. In fact, in older adults, multimorbidity, the co‐occurrence of two or more chronic conditions in the same individual, is the rule rather than the exception.^13^ Diseases cluster in the same individual following specific patterns due to shared risk factors or underlying pathophysiological mechanisms.^14^ Individuals with certain multimorbidity patterns have distinct sociodemographic profiles, health, and functional status.^15^ Moreover, these patterns have been linked to an increased risk of dementia^16^ and differentially steer individual trajectories across the cognitive continuum.^17^ Whether the blood concentration of AD biomarkers is affected by the presence of distinct multimorbidity patterns is crucial for accurate diagnosis, optimizing trial enrollment, and guiding appropriate drug prescription, ultimately providing robust clinical guidance.

In the present study, we aimed to cross‐sectionally investigate the variations of the circulating level of several AD blood biomarkers in relation to multimorbidity and specific multimorbidity patterns in a community‐based cohort of > 2200 cognitively unimpaired older adults.

## 2 METHODS

### 2.1 Study population

Data were gathered from the Swedish National Study on Aging and Care in Kungsholmen (SNAC‐K), an ongoing population‐based study.^18^ SNAC‐K includes community‐dwelling and institutionalized adults aged ≥ 60 years, living in the Kungsholmen district of Stockholm, Sweden. At baseline (2001–2004), a random sample of 5111 people from 11 age cohorts were invited to participate in the study. Of the 4590 eligible individuals, 3363 were examined (participation rate: 73%). Younger participants (age < 78 years) were followed every 6 years, and the older (age ≥ 78 years) every 3 years. In the present study, we identified 2290 persons who were dementia free and had available blood biomarker data at baseline. Figure S1 in supporting information depicts the flowchart of study participation.

All phases of SNAC‐K and the use of inpatient registry data were approved by the ethics committee at Karolinska Institutet (KI) and the Regional Ethical Review Board in Stockholm (Dnrs: KI 01‐114, 04‐929/3, Ö26‐2007, 2009/595‐32, 2010/447‐31/2, 2013/828‐31/3 and 2016/730‐31/1). All participants provided written informed consent. The results of this study are reported following the Strengthening the Reporting of Observational Studies in Epidemiology (STROBE) recommendations.

### 2.2 Data collection

At each study wave, SNAC‐K participants undergo a comprehensive clinical and functional assessment by trained physicians, nurses, and psychologists, following standard procedures. Home visits are carried out for those who agree to participate but are unable to reach the research center.

### 2.3 Covariates

Participants’ demographic information (i.e., age, sex, and education) was collected during nurses’ interviews. Educational attainment was categorized as elementary, high school, and university or higher, indicating the highest level of education attained. The Mini‐Mental State Examination (MMSE) score was used as a measure of global cognition. The clinical diagnosis of dementia followed a three‐step procedure and is performed in keeping with the Diagnostic and Statistical Manual of Mental Disorders, Fourth Edition (DSM‐IV) criteria.^19^ DNA was extracted from peripheral blood samples and apolipoprotein E (*APOE*) alleles were genotyped. Based on the *APOE* alleles, participants were categorized as ε4 carriers (at least one ε4 allele) versus non‐carriers. Creatinine was measured at baseline, and the estimated glomerular filtration rate (i.e., eGFR) was calculated using the 2009 Chronic Kidney Disease Epidemiology Collaboration (CKD‐EPI) equation.

### 2.4 Chronic disease assessment

Details on definition and classifications of diseases are reported elsewhere.^20^ Briefly, physicians collected information on diagnoses via physical examination, medical history, self‐reported information, and/or proxy interviews. Clinical parameters, lab tests, medication, and inpatient and outpatient care data were also used to identify specific conditions. All diagnoses were coded according to the International Classification of Diseases, Tenth Revision (ICD‐10) and classified into 60 chronic disease categories in accordance with a clinically driven methodology. Drugs were classified and coded in accordance with the Anatomical Therapeutic Chemical (ATC) classification. Chronic diseases were considered at baseline. Prescribed medications were used to adjudicate certain diagnoses, but only in instances in which the drug was unequivocally indicative of the underlying condition.

**RESEARCH IN CONTEXT**

1. **Systematic review**: The literature research showed that blood biomarkers of Alzheimer's disease (AD) are emerging as reliable indicators of AD pathology and neurodegeneration and accurately predict future cognitive decline, including the onset of dementia. However, studies show that a higher number of co‐occurring chronic diseases and specific conditions like chronic kidney disease, obesity, diabetes, hypertension, and may impact blood biomarker concentration.
2. **Interpretation**: While previous research has investigated how multimorbidity and individual chronic conditions alter the circulating levels of blood biomarkers of AD, this is the first study examining the influence of specific combinations of diseases (i.e., multimorbidity patterns) on a large set of blood biomarkers of AD within a community setting of cognitively intact adults. Findings show that blood biomarkers of AD vary depending on the number of chronic diseases and specific patterns of multimorbidity. Results remain unchanged after excluding participants who develop dementia during 15 years of follow‐up.
3. **Future directions**: Future studies are needed to better understand the interplay between biomarkers of AD, somatic conditions, and their relationship with future dementia development as well as the potential need of tailored cutoffs especially in older adults.

### 2.5 Blood biomarkers of AD

AD biomarkers were quantified in peripheral blood samples provided by the participants at baseline. We measured amyloid beta (Aβ42 and Aβ40), phosphorylated tau at residual threonine 181 (p‐tau181) and 217 (p‐tau217), total tau (t‐tau), neurofilament light chain (NfL), and glial fibrillary acidic protein (GFAP). Upon blood centrifugation, serum aliquots were stored at −80°C at the KI Bio Bank until time of analysis. The concentrations of the biomarkers were measured using ultrasensitive single‐molecule array (Simoa) technology at the Affinity Proteomics Stockholm Unit (SciLifeLab). The Quanterix instruments provide a relative average enzyme per bead (AEB) values for calibrator, controls, and samples for each protein; Quanterix SR‐X software automatically performs curve‐fitting, extrapolation of concentrations, and graphical representation of the results. Simoa Neuro 3‐plex A Kit (cat. 101995; lot. 503659) was used for Aβ isoforms and t‐tau, Simoa pTau‐181 Advantage V2 Kit for p‐tau181 (cat. 103714; lot. 503703), and Simoa Neuro 2‐plex B Kit (cat. 103520; lot. 503409) for NfL and GFAP. P‐tau217 was quantified using the commercial assay Simoa ALZpath p‐tau217 Advantage PLUS (cat. 104570; lot. 504307) developed for the Quanterix HD‐X system and validated for SR‐X. Single‐value imputation was used to replace data below the limit of detection (LOD) with a value of 0; in total, we imputed six measures for Aβ42, fifteen for both p‐tau181 and t‐tau, and five for p‐tau217.

### 2.6 Statistical analysis

We used the Kruskal–Wallis test to compare biomarker concentrations between groups. Blood biomarkers were transformed into *z* scores based on baseline mean and standard deviation of the study population, to allow comparison between coefficients.

Quantile regression models on the 50th (median) percentile were used to examine the association between the number of chronic diseases and the level of AD blood biomarkers. The number of chronic diseases was modelled using restricted cubic splines with three data‐driven knots (at 1, 3, and 7) and the analyses were adjusted for age, sex, and education.

Quantile regression models on the median were also used to examine the association between multimorbidity patterns and the blood concentration of AD biomarkers, adjusting for age, sex, and education. Latent class analysis (LCA) was used to identify patterns of multimorbidity.^21^ This analysis included 37 groups of chronic conditions, each with a baseline prevalence of at least 2%. The optimal number of classes was determined using the adjusted Bayesian information criterion (ABIC) and the theoretical interpretability by geriatric medicine experts. Participants were assigned to the class with the highest posterior probability of membership. Two measures were used to identify overexpressed conditions within these patterns: the observed/expected (O/E) ratio and disease exclusivity. The O/E ratio was calculated by dividing the prevalence of a condition within a pattern by its prevalence in the overall multimorbid sample. Disease exclusivity was defined as the proportion of participants with the condition within the pattern compared to the total number of participants with the condition in the multimorbid sample. Conditions were considered to characterize and name the patterns if they had an O/E ratio of at least 2 and an exclusivity of at least 25%.

We performed sensitivity and stratified analyses by: (1) repeating the analyses by excluding those who developed dementia over the follow‐up period; (2) restricting the analyses to individuals with multimorbidity, using the *unspecific* group as the reference; (3) repeating the analyses with further adjustment for kidney function (i.e., eGFR). Interactions with age and sex were tested, and stratified analyses were conducted.

A two‐tailed *p* value < 0.05 was considered statistically significant in all analyses. The statistical analyses were performed using Stata version 18 (StataCorp); GraphPad Prism 9 for graphical representations; and R Statistical Software version 4.3.2 (packages: “poLCA”) for LCA.^21^

## 3 RESULTS

Baseline sample characteristics are shown in Table 1. The mean age of the population was 72 years, 62% were women, and 36% had a university level of education. Overall, 29% of the sample carried at least one *APOE* ε4 allele, and the mean MMSE score was 28. Of the 2290 participants, all of whom were cognitively unimpaired, 85% (*N* = 1939) were affected by multimorbidity. The following multimorbidity patterns were identified: psychiatric/respiratory/musculoskeletal (*N* = 359), anemia/sensory impairment (*N* = 405), cardiometabolic/inflammatory (*N* = 225). The remaining individuals (*N* = 950) were grouped in a cluster in which no disease was overexpressed, in relation to the overall population prevalence, and thus was named “unspecific multimorbidity.” This pattern was chosen as reference group as it includes the youngest individuals, with the lowest mean number of diseases and the lowest 6‐ and 12‐year mortality.^14^ Table S1 in supporting information reports the list of chronic diseases included in each pattern, their prevalence within the pattern, the observed to expected ratio, and their exclusivity.

**TABLE 1:** Baseline characteristics of the study population by multimorbidity patterns.

	Total	Non‐multimorbid	Unspecific	Psychiatric, respiratory & MSK	Anemia & sensory impairment	Cardiometabolic & inflammatory
	*N* = 2290	*N *= 351	*N* = 950	*N* = 359	*N* = 405	*N* = 225
**Age** (years, mean ± SD)	72.4 (± 10.5)	64.5 (± 6.4)	69.8 (± 8.6)	70.6 (± 9.2)	80.7 (± 9.4)	83.3 (± 8.9)
**Women,** (*n*, %)	1410 (61.6%)	188 (53.6%)	556 (58.5%)	270 (75.2%)	264 (65.2%)	132 (58.7%)
**University level education** (*n*, %)	827 (36.1%)	186 (53.0%)	355 (37.4%)	128 (35.6%)	114 (28.2%)	44 (19.6%)
**MMSE** (mean, ± SD)	28.6 (± 1.9)	29.2 (± 1.6)	28.9 (1.5)	28.8 (1.7)	27.9 (± 2.2)	27.4 (2.9)
**Chronic diseases** (mean, ± SD)	3.8 (± 2.4)	0.8 (0.4)	3.2 (1.2)	4.4 (1.8)	5.0 (1.8)	7.7 (2.2)
***APOE* ε4** (at least one ε4 allele, *n*, %)	653 (29.4%)	89 (26.2%)	287 (31.2%)	120 (34.5%)	106 (26.6%)	51 (23.8%)

*Note*: Missing: 1 in education, 69 in *APOE* genotype, 4 in MMSE.

Abbreviations: *APOE*, apolipoprotein E; MMSE, Mini‐Mental State Examination; MSK, musculoskeletal; SD, standard deviation.

Participants in the anemia/sensory impairment and in the cardiometabolic/inflammatory patterns were older, less educated, and with a higher mean number of chronic diseases than those in the unspecific one. Individuals in the psychiatric/respiratory/musculoskeletal pattern were mostly women (75.2%) with a similar mean age as those in the unspecific pattern.

**FIGURE 1:**
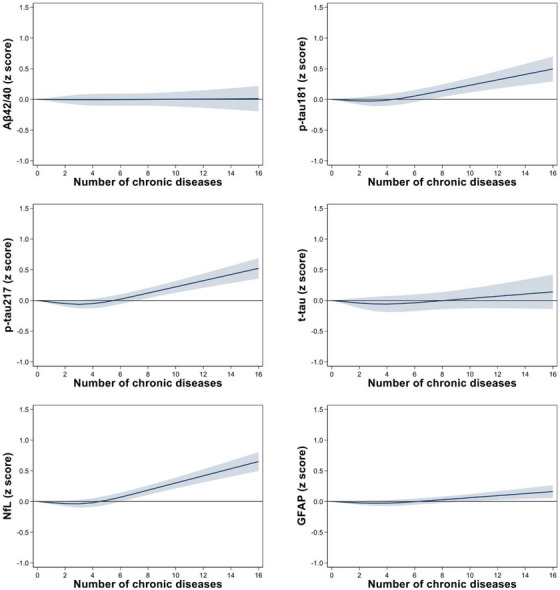
Concentration of blood biomarkers of Alzheimer's disease (*z* score) in relation to the number of chronic diseases at baseline. Estimates are derived from quantile regression models on the 50th (median) percentile adjusted for age, sex, and education. The number of chronic diseases is modelled using restricted cubic splines with three data driven knots (at 1, 3, and 7). Zero chronic disease was chosen as the reference for graphical representation. Aβ, amyloid beta; GFAP, glial fibrillary acidic protein; NfL, neurofilament light chain; p‐tau, phosphorylated tau; t‐tau, total tau.

Figure 1 shows the concentration of blood biomarkers (*z* score) in relation to the number of chronic diseases at baseline adjusted for age, sex, and education. The levels of p‐tau181, p‐tau217, NfL, and GFAP increased with increasing number of conditions whereas Aβ42/40 ratio and t‐tau levels did not substantially differ.

**FIGURE 2:**
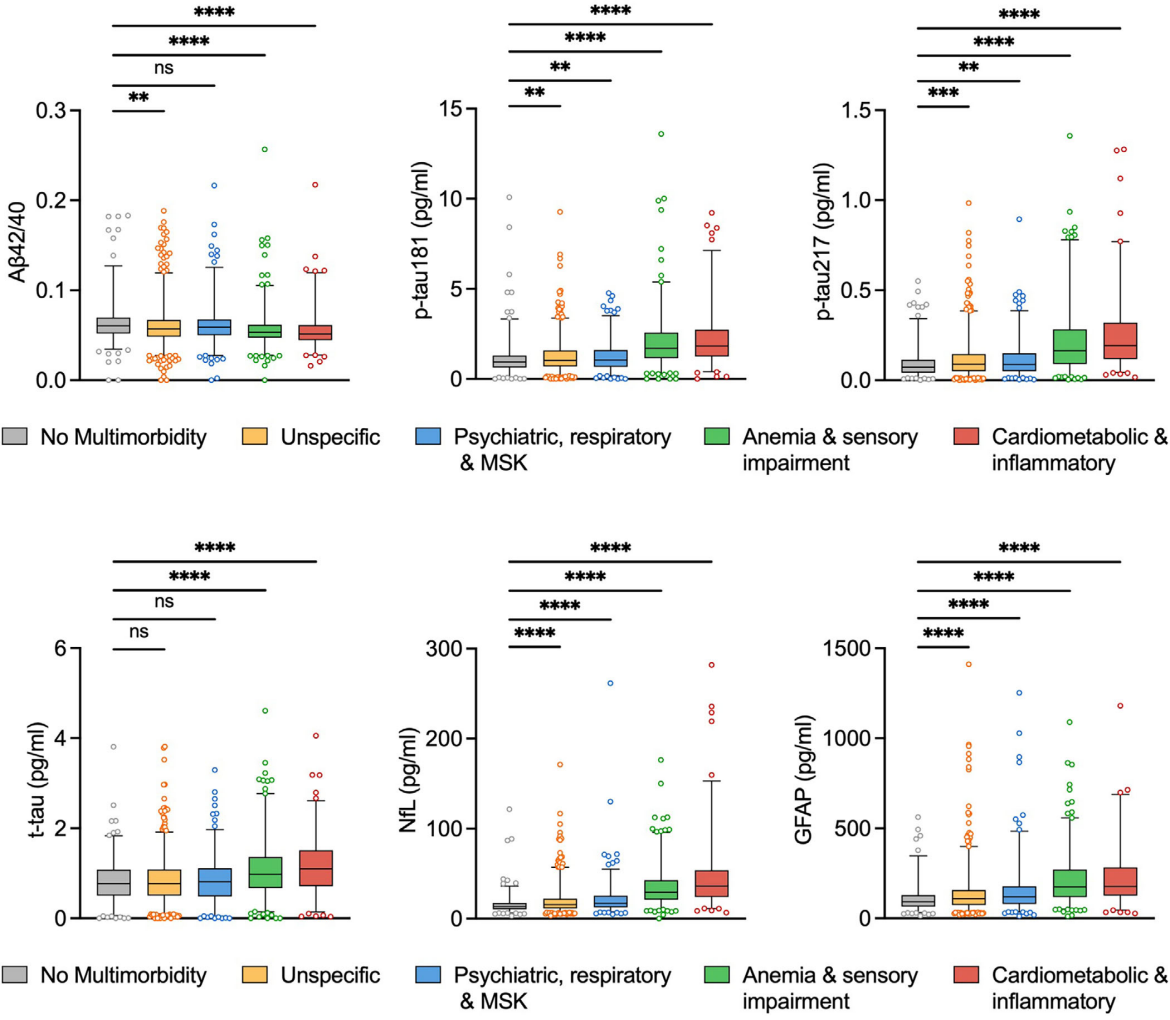
Distribution of the concentration of blood biomarkers of Alzheimer's disease by multimorbidity patterns. Box plots show the median (central line) and interquartile range (box) as well as the 2.5th and 97.5th percentiles (whiskers). *p* values are derived from Kruskal–Wallis test; ns: non‐significant, *: < 0.05; **: < 0.01; ***: < 0.001; ****: < 0.0001. Some outliers were not represented for graphical purposes (*n* = 2 for Aβ42/40 ratio, p‐tau181, t‐tau, and NfL; *n* = 7 for GFAP). Aβ42/40, amyloid beta 42/40; GFAP, glial fibrillary acidic protein; MSK, musculoskeletal; NfL, neurofilament light chain; p‐tau181, phosphorylated‐tau181; t‐tau, total‐tau.

When blood biomarkers were analyzed across specific multimorbidity patterns, the Aβ42/40 ratio was slightly lower while p‐tau181, p‐tau217, t‐tau, NfL, and GFAP levels were higher in the anemia/sensory impairment and the cardiometabolic/inflammatory patterns compared to those without multimorbidity (Figure 2).

**FIGURE 3:**
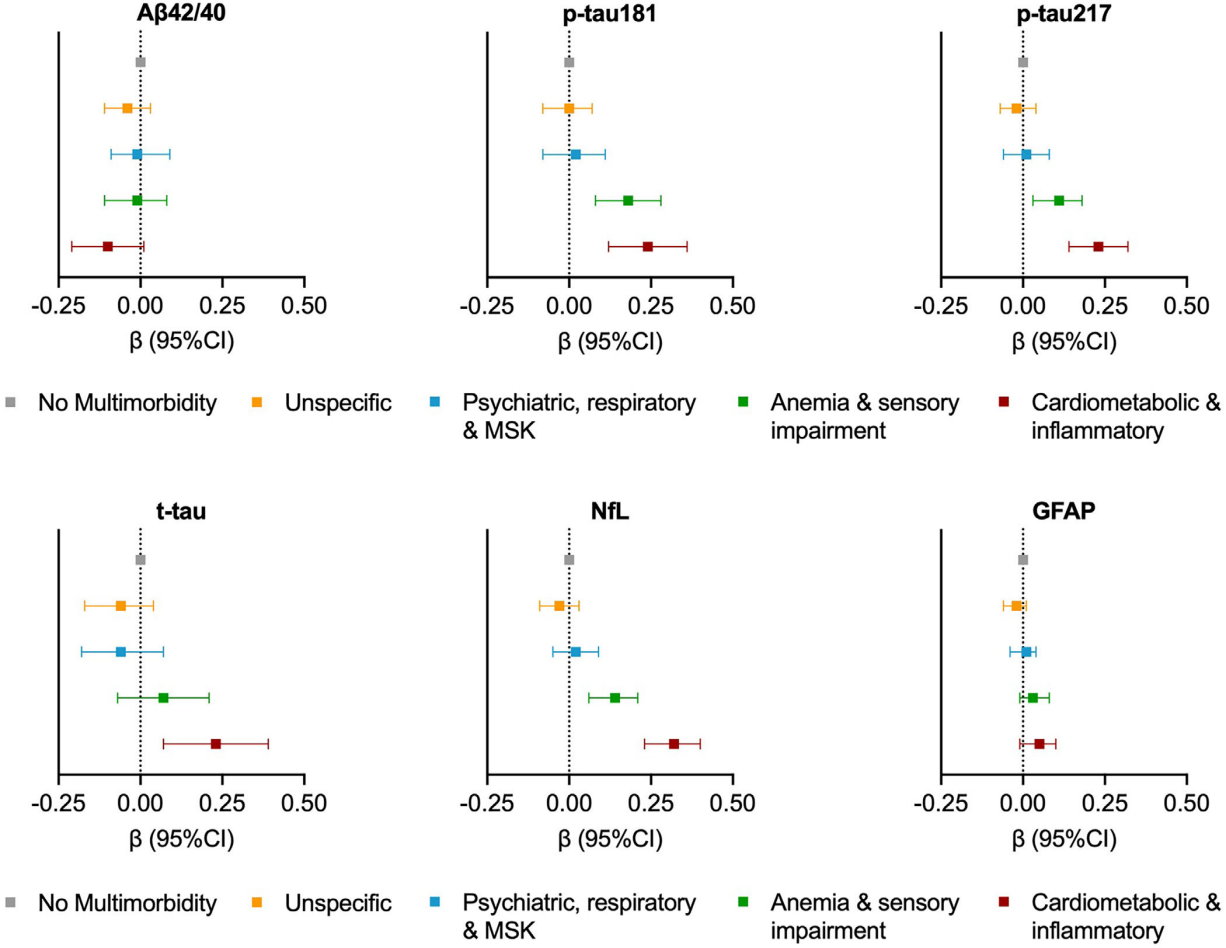
Associations between multimorbidity patterns and the concentration of blood biomarkers of Alzheimer's disease. β and 95% CIs are derived from quantile regression models on the 50th (median) percentile, adjusted for age, sex and education. Aβ, amyloid beta; CI, confidence interval; GFAP, glial fibrillary acidic protein; MSK, musculoskeletal; NfL, neurofilament light chain; p‐tau, phosphorylated tau; t‐tau, total tau.

In multi‐adjusted quantile regression models (Figure 3 and Table S2 in supporting information) p‐tau181, p‐tau 217, and NfL levels were all significantly higher in the anemia/sensory impairment and cardiometabolic/inflammatory patterns compared to the non‐multimorbid one. Notably, NfL levels were significantly higher in the cardiometabolic/inflammatory pattern compared to the other two patterns (NfL levels in cardiometabolic/inflammatory: β 0.32; 95% confidence interval [CI]: 0.23–0.40; in anemia/sensory impairment: β: 0.14; 95% CI: 0.06–0.21; in psychiatric/respiratory/musculoskeletal: β: 0.02; 95% CI: −0.05, 0.09).

We also performed sensitivity and stratified analyses. (1) After excluding those individuals who developed dementia during the follow‐up (*N* = 364), the findings remained similar to the main ones with the exception of the association with GFAP that became no more statistically significant for disease count (Tables S3 and S4 in supporting information). (2) Table S5 in supporting information shows the multi‐adjusted quantile regression model when we restricted the analyses to the multimorbid group only, with the unspecific group as the reference. Results were similar to the main ones, with the exception of the association with GFAP, which became statistically significant. (3) Most of the results did not change significantly after further adjustment for kidney function (eGFR; Table S6 in supporting information). The only exception was the association between the cardiometabolic/inflammatory pattern and GFAP, which became non‐significant.

Tables S7 and S8 in supporting information show the associations between AD blood biomarkers and multimorbidity patterns by age groups and sex. Considering the cardiometabolic/inflammatory pattern p‐tau181, t‐tau, and GFAP levels were higher in the younger participants, while NfL was higher in the oldest ones. P‐tau217 levels were higher in the oldest in the anemia/sensory impairment pattern compared to the unspecific pattern. Men in the anemia/sensory impairment and the cardiometabolic/inflammatory patterns had generally higher levels of p‐tau181, p‐tau 218, t‐tau, NfL, and GFAP compared to men in the unspecific pattern.

## 4 DISCUSSION

This study, based on a large cohort of cognitively unimpaired community dwelling older adults, shows that the circulating level of AD blood biomarkers vary depending on the number of chronic diseases and patterns of multimorbidity. We observed that: (1) p‐tau181, p‐tau217, NfL, and GFAP levels increased along with increasing numbers of chronic diseases; and (2) p‐tau181, p‐tau217, t‐tau, NfL, and GFAP levels were significantly higher in individuals in the anemia/sensory impairment and cardiometabolic/inflammatory multimorbidity patterns compared to those in the unspecific one. Results remained unchanged after excluding participants who developed dementia during the follow‐up. While previous research has investigated how multimorbidity and individual chronic conditions alter the circulating levels of blood biomarkers of AD, this is the first study examining the influence of specific combinations of diseases (i.e., multimorbidity patterns) on a large set of blood biomarkers of AD within a community setting of cognitively intact adults.

A recent study from our group using the same cohort found that a greater disease burden was linked with a variation in the levels of several AD blood biomarkers.^8^ Similar results emerged in other population‐based studies, including the Mayo Clinic Study of Aging in which plasma levels of Aβ40, Aβ42, the Aβ42/40 ratio, p‐tau181, p‐tau217, t‐tau, and NfL increased with higher Charlson Comorbidity Index scores.^9^ ^11^ Similarly, in the Multimodal Interventions to Delay Dementia and Disability in China (MIND‐China) study, an increasing number of chronic diseases was linked with elevated levels of plasma Aβ40, Aβ42, and NfL.^22^ In our study, we observed that p‐tau181, p‐tau217, NfL, and GFAP levels were all influenced by the number of chronic conditions, with NfL showing the greatest increase. This is not unexpected, as NfL is a non‐specific marker of neurodegeneration, likely influenced by several somatic conditions.^10^, ^23^, ^24^, ^25^ The result on the two p‐tau isoforms is particularly interesting because they accurately reflect AD pathology and, as such, their interpretation needs to be explored. In these contexts, as in primary care, individuals are more heterogeneous and often experience a higher frequency and diversity of chronic conditions compared to those included in memory clinics.

We further expand this knowledge by looking not only at multimorbidity, but at multimorbidity patterns and their link with AD biomarker concentrations. While two previous studies from MIND‐China looked at multimorbidity patterns and AD blood biomarkers, direct comparisons are hindered due to their inclusion of individuals with dementia. In another study, Ren et al. found that the metabolic and degenerative ocular multimorbidity patterns were associated with elevated plasma NfL levels, while the cardiac‐musculoskeletal pattern was linked to increased plasma Aβ42 levels.^22^ In a study by Liu et al., they reported that cardiometabolic multimorbidity was significantly associated with higher plasma levels of Aβ40, Aβ42, and NfL.^26^ Based on our findings, individuals with anemia and sensory impairment and those with cardiometabolic and inflammation had higher concentrations of all biomarkers with the only exception of the Aβ42/40 ratio. It is possible that altered biomarker concentrations reflect an increased pathological burden, in which specific multimorbidity patterns exacerbate brain damage, leading to elevated biomarker levels.^27^, ^28^ Individuals within the cardiometabolic/inflammatory pattern showed the highest levels of p‐tau181, p‐tau217, t‐tau, NfL, and GFAP, nearly double compared to the levels observed in those within the anemia/sensory impairment pattern, and significantly higher than the levels observed within the psychiatric/respiratory/musculoskeletal pattern. Several individual cardiovascular conditions, including atrial fibrillation,^8^ heart failure,^29^ and stroke,^11^ have previously been linked to altered blood biomarker levels. For instance, Mielke et al. found higher plasma concentrations of p‐tau181 and p‐tau217 in individuals with hypertension, stroke, and myocardial infarction.^11^ Therefore, it is not surprising that we observed these conditions clustering in the same pattern and being strongly associated with the highest levels of blood biomarkers. A close heart–brain connection is well known, and cardiovascular diseases are recognized to increase the risk of dementia.^30^ This likely explains why individuals with a cardiometabolic profile had elevated biomarker levels, potentially reflecting greater brain pathology related to those health conditions. The co‐occurrence of cardiometabolic and inflammatory diseases may stem from shared underlying pathophysiological mechanisms, such as chronic inflammation, oxidative stress, and vascular dysfunction, all of which are known to accelerate neurodegeneration and increase dementia risk.^31^ Chronic inflammatory diseases are linked to an increased risk of dementia, likely due to prolonged systemic inflammation with an alteration of the blood–brain barrier contributing to the accumulation of brain pathology.^32^ In addition, persistent low‐grade inflammation can accelerate oxidative stress and neuronal damage, promoting the accumulation of AD pathology. When cardiometabolic and inflammatory diseases co‐occur, they may act synergistically, with detrimental effects on the brain that lead to higher levels of AD biomarkers. These findings align with previous work, in which individuals presenting with a cardiovascular pattern had a greater hazard of developing dementia compared to those with an unspecific disease pattern.^16^ This association was particularly pronounced when systemic inflammation was present, suggesting that the interplay between cardiometabolic and inflammatory conditions may exacerbate AD neuropathology. Also, an association between chronic inflammatory diseases, such as autoimmune diseases, and cognitive impairment has been demonstrated.^33^ The combination of cardiovascular dysfunction and chronic inflammation could alter blood–brain barrier permeability, promote tau phosphorylation, and accelerate neuronal injury, thereby elevating AD biomarkers. Participants in the anemia/sensory impairment pattern had also significantly higher levels of biomarkers compared to the psychiatric/respiratory/musculoskeletal and unspecific patterns.

We have previously reported anemia as a condition that potentially alters the levels of several blood biomarkers.^8^ Anemia contributes to hypoxia, which can be associated with increased oxidative stress, inflammation, and the disruption of the blood–brain barrier, all of which may exacerbate brain pathology.^34^ In addition to hypoxia, anemia might also influence AD biomarker levels through other pathways, such as impaired iron metabolism,^35^ which can contribute to abnormal protein aggregation and inflammation. It is interesting to note that in this study, anemia clustered with sensory impairment. While this combination might appear surprising at first glance, these two conditions probably tend to co‐occur as a reflection of biological changes associated with aging. Hemoglobin concentration, mean corpuscular volume, red blood cell count distribution, and metabolism change with human aging^36^, ^37^, ^38^ and age‐related hearing loss (presbycusis) is a common problem linked to aging.^34^ This is further supported by the fact that individuals in this cluster are particularly old.

Interestingly, we observed a similar association between blood biomarkers of AD and both number of diseases and the anemia/sensory impairment and the cardiometabolic/inflammatory patterns even after excluding individuals who developed dementia during a 15‐year follow‐up, suggesting that these conditions may alter biomarker concentrations even in those without impending dementia. One hypothetical explanation could be that specific multimorbidity patterns may contribute to neurodegenerative damage several years before dementia onset. It has been estimated that neuropathological lesions could be present as long as 30 years before the onset of clinical AD.^35^ Peripheral factors may also play a role, such as altered protein redistribution, impaired metabolism, or reduced clearance, as seen for conditions like chronic kidney disease.^39^, ^40^ These peripheral alterations could contribute to changes in biomarker levels independently of brain pathology. An important consideration is that in our analyses, most results remained unchanged even after adjusting for kidney function (eGFR). Given the decline in kidney function with aging,^41^ adjusting for glomerular filtration rate has been recommended when using NfL to define neurodegeneration status.^42^ On the other hand, eGFR formulas are less accurate in the oldest‐old compared to the younger‐old. Consequently, participants in the anemia and sensory impairment as well as those in the cardiometabolic and inflammation pattern may exhibit higher levels of these biomarkers due to their very advanced age, which could lead to an overestimation of their renal function.

Key strengths of the current study include the large population‐based sample, including extensive in‐person evaluations of comorbidities. In addition, we had the chance to analyze an extensive panel of AD biomarkers, including p‐tau217, which is currently considered the most accurate predictor of AD development.^12^ Some limitations need to be acknowledged. In our study, AD biomarkers were measured in serum, which can affect their bioavailability compared to plasma. For instance, Aβ levels, like other protein biomarkers, may exhibit greater stability in plasma. However, serum biomarkers of AD have been previously used to predict dementia development^43^ and have shown a good correlation with plasma and cerebrospinal fluid biomarkers and a similar diagnostic accuracy.^44^, ^45^ Finally, this study is based on a population‐based sample of older adults residing in an urban area of central Stockholm, Sweden. As such, the cohort lacks ethnic diversity as well as socioeconomic heterogeneity, and caution is warranted when generalizing these findings to more ethnically and socioeconomically diverse populations.

## 5 CONCLUSIONS

In conclusion, our study provides new insights into how multimorbidity patterns are associated with variations in AD blood biomarkers. We found that specific patterns, such as anemia/sensory impairment and cardiometabolic/inflammatory patterns, are linked to higher levels of AD biomarkers. Future studies are needed to better untangle the interplay among biomarkers of AD, somatic conditions, and their relationship to future dementia development especially in the oldest fragment of the population. Furthermore, tailored cutoffs may be crucial to avoid misclassification and false positives before extending biomarker measurement in primary care and in community settings.

## AUTHOR CONTRIBUTIONS

Alessandra Marengoni, Giulia Grande, and Davide Liborio Vetrano contributed to the conception and design of the study. Claudia Fredolini and Matilda Dale conducted the biomarker analyses. Martina Valletta, Caterina Gregorio, and Giulia Grande conducted statistical analyses. Alessandra Marengoni and Giulia Grande conducted the literature search. All authors contributed to interpretation of the results. Alessandra Marengoni and Giulia Grande drafted the first version of the manuscript. All authors critically revised the manuscript for important intellectual content. All authors made a significant contribution to the research and the development of the manuscript and approved the final version for publication.

## FUNDING

Data collection of the Swedish National Study on Aging and Care (SNAC‐K) was supported by the Swedish Research Council (current grant: 2021‐00178); the Swedish Ministry of Health and Social Affairs; and the participating County Councils and Municipalities. This work was further supported by Stiftelsen Sigurd och Elsa Goljes minne (MV, project nr: LA2023‐0113), Hjärnfonden (postdoc stipend to GG; 2021‐0025) and Gamla Tjänarinnor foundation (GG 2021‐01235); DLV was supported by the Swedish Research Council (project number 2021‐03324) and the Karolinska Institutet Strategic Research Area in Epidemiology and Biostatistics (SFOepi) in 2021. Margaretha af Ugglas’ foundation (BW).

## CONFLICT OF INTEREST STATEMENT

The authors declare no conflicts of interest. Author disclosures are available in the Supporting Information.

## CONSENT STATEMENT

All participants provided written informed consent to participate in the study.

## ROLE OF THE FUNDING SOURCE

The funders had no role in study design, data collection, data analyses, data interpretation, or writing of the report.

## Supporting information



Supporting information

Supporting information
