# Supplementary material for: Multimorbidity patterns and blood biomarkers of Alzheimer's disease in community‐dwelling cognitively unimpaired older adults
Source: Alzheimers Dement. 2025 Jun 22;21(6):e70411. doi: 10.1002/alz.70411 (PMC12183106; doi:10.1002/alz.70411)

**Supplementary material**

**Table S1.** Description of multimorbidity patterns

| **Multimorbidity pattern** | **Chronic condition** | **Prevalence within the pattern (%)** | **Exclusivity (%)** | **O/E** |
| --- | --- | --- | --- | --- |
| **Unspecific**  **(n=950)** | Anemia | 1 | 5 | 0.10 |
|  | Asthma | 4 | 23 | 0.49 |
|  | Atrial fibrillation | 4 | 18 | 0.39 |
|  | Autoimmune diseases | 3 | 28 | 0.61 |
|  | Blindness, visual loss | <1 | <1 | <0.01 |
|  | Bradycardias and conduction diseases | <1 | 8 | 0.18 |
|  | COPD, emphysema and chronic bronchitis | 2 | 14 | 0.31 |
|  | Cardiac valve diseases | 1 | 15 | 0.33 |
|  | Cataract and other lens diseases | 1 | 6 | 0.13 |
|  | Cerebrovascular diseases | 4 | 22 | 0.48 |
|  | Chronic kidney diseases | 23 | 28 | 0.61 |
|  | Colitis and related diseases | 2 | 7 | 0.16 |
|  | Deafness and hearing loss | 3 | 13 | 0.28 |
|  | Depression and mood diseases | 2 | 9 | 0.19 |
|  | Diabetes | 9 | 44 | 0.94 |
|  | Dorsopathies | 3 | 20 | 0.44 |
|  | Dyslipidemia | 68 | 59 | 1.27 |
|  | Esophagus, stomach and duodenum diseases | 3 | 30 | 0.65 |
|  | Glaucoma | 1 | 7 | 0.16 |
|  | Heart failure | 1 | 4 | 0.09 |
|  | Hypertension | 86 | 53 | 1.14 |
|  | Inflammatory arthropathies | 2 | 16 | 0.34 |
|  | Ischemic heart diseases | 11 | 32 | 0.68 |
|  | Migraine and facial pain syndromes | 2 | 37 | 0.79 |
|  | Neurotic, stress-related and somatoform diseases | <1 | <1 | <0.01 |
|  | Obesity | 20 | 65 | 1.41 |
|  | Osteoarthritis and other degenerative joint diseases | 12 | 38 | 0.83 |
|  | Osteoporosis | <1 | 0 | <0.01 |
|  | Other MSK joint diseases | 5 | 35 | 0.77 |
|  | Other cardiovascular diseases | 1 | 11 | 0.23 |
|  | Other eye diseases | <1 | 1 | 0.01 |
|  | Other genitourinary diseases | 3 | 45 | 0.97 |
|  | Other neurological diseases | 1 | 20 | 0.44 |
|  | Prostate diseases | 4 | 44 | 0.95 |
|  | Sleep diseases | 2 | 43 | 0.93 |
|  | Solid neoplasms | 8 | 37 | 0.81 |
|  | Thyroid diseases | 7 | 30 | 0.65 |
| **Anemia & sensory impairment**  **(n=359)** | **Anemia** | **31** | **54** | **2.40** |
|  | Asthma | 1 | 4 | 0.16 |
|  | Atrial fibrillation | 9 | 20 | 0.86 |
|  | Autoimmune diseases | 6 | 26 | 1.14 |
|  | **Blindness, visual loss** | **13** | **68** | **3.01** |
|  | Bradycardias conduction diseases | <1 | 0 | <0.01 |
|  | COPD, emphysema and chronic bronchitis | 2 | 9 | 0.41 |
|  | Cardiac valve diseases | 3 | 21 | 0.91 |
|  | **Cataract and other lens diseases** | **21** | **77** | **3.39** |
|  | Cerebrovascular diseases | 12 | 34 | 1.49 |
|  | Chronic kidney diseases | 58 | 34 | 1.52 |
|  | Colitis and related diseases | 16 | 30 | 1.34 |
|  | **Deafness and hearing loss** | **27** | **55** | **2.41** |
|  | Depression and mood diseases | 5 | 11 | 0.50 |
|  | Diabetes | 9 | 20 | 0.88 |
|  | Dorsopathies | 6 | 17 | 0.75 |
|  | Dyslipidemia | 35 | 15 | 0.67 |
|  | Esophagus, stomach and duodenum diseases | 3 | 14 | 0.64 |
|  | **Glaucoma** | **20** | **72** | **3.17** |
|  | Heart failure | 2 | 3 | 0.15 |
|  | Hypertension | 77 | 23 | 1.01 |
|  | Inflammatory arthropathies | 7 | 33 | 1.47 |
|  | Ischemic heart diseases | 15 | 21 | 0.92 |
|  | Migraine and facial pain syndromes | 1 | 10 | 0.45 |
|  | Neurotic, stress-related and somatoform diseases | 1 | 8 | 0.34 |
|  | Obesity | 6 | 10 | 0.45 |
|  | Osteoarthritis and other degenerative joint diseases | 13 | 19 | 0.85 |
|  | Osteoporosis | 12 | 37 | 1.62 |
|  | Other MSK joint diseases | 6 | 19 | 0.86 |
|  | Other cardiovascular diseases | 2 | 14 | 0.60 |
|  | **Other eye diseases** | **19** | **73** | **3.23** |
|  | Other genitourinary diseases | 4 | 31 | 1.36 |
|  | Other neurological diseases | 3 | 32 | 1.42 |
|  | Prostate diseases | 6 | 29 | 1.29 |
|  | Sleep diseases | <1 | 1 | 0.07 |
|  | Solid neoplasms | 17 | 38 | 1.68 |
|  | Thyroid diseases | 9 | 18 | 0.81 |
| **Cardiometabolic & inflammatory**  **(n=225)** | **Anemia** | **32** | **33** | **2.48** |
|  | Asthma | 8 | 14 | 1.10 |
|  | **Atrial fibrillation** | **45** | **57** | **4.30** |
|  | **Autoimmune diseases** | **11** | **27** | **2.08** |
|  | **Blindness, visual loss** | **10** | **31** | **2.36** |
|  | **Bradycardias conduction diseases** | **14** | **81** | **6.18** |
|  | **COPD, emphysema and chronic bronchitis** | **16** | **36** | **2.75** |
|  | **Cardiac valve diseases** | **13** | **60** | **4.58** |
|  | Cataract and other lens diseases | 6 | 12 | 0.90 |
|  | **Cerebrovascular diseases** | **21** | **36** | **2.74** |
|  | Chronic kidney diseases | 72 | 25 | 1.88 |
|  | Colitis and related diseases | 22 | 24 | 1.83 |
|  | Deafness and hearing loss | 21 | 24 | 1.84 |
|  | Depression and mood diseases | 11 | 15 | 1.11 |
|  | **Diabetes** | **23** | **31** | **2.33** |
|  | Dorsopathies | 11 | 19 | 1.48 |
|  | Dyslipidemia | 42 | 10 | 0.78 |
|  | Esophagus, stomach, and duodenum diseases | 7 | 19 | 1.42 |
|  | Glaucoma | 8 | 16 | 1.24 |
|  | **Heart failure** | **75** | **92** | **7.00** |
|  | Hypertension | 67 | 12 | 0.89 |
|  | **Inflammatory arthropathies** | **13** | **36** | **2.71** |
|  | **Ischemic heart diseases** | **55** | **43** | **3.27** |
|  | Migraine and facial pain syndromes | 5 | 25 | 1.90 |
|  | Neurotic, stress-related and somatoform diseases | 3 | 11 | 0.83 |
|  | Obesity | 14 | 13 | 0.96 |
|  | Osteoarthritis and other degenerative joint diseases | 18 | 16 | 1.18 |
|  | Osteoporosis | 13 | 23 | 1.75 |
|  | Other MSK joint diseases | 10 | 20 | 1.51 |
|  | **Other cardiovascular diseases** | **19** | **66** | **5.02** |
|  | Other eye diseases | 8 | 18 | 1.38 |
|  | Other genitourinary diseases | 3 | 15 | 1.17 |
|  | Other neurological diseases | 1 | 8 | 0.64 |
|  | Prostate diseases | 8 | 21 | 1.62 |
|  | Sleep diseases | 3 | 15 | 1.13 |
|  | Solid neoplasms | 10 | 13 | 0.96 |
|  | Thyroid diseases | 13 | 15 | 1.13 |
| **Psychiatric, respiratory & MSK (n=359)** | Anemia | 6 | 8 | 0.47 |
|  | **Asthma** | **24** | **59** | **3.31** |
|  | Atrial fibrillation | 3 | 6 | 0.32 |
|  | Autoimmune diseases | 5 | 19 | 1.04 |
|  | Blindness, visual loss | <1 | 1 | 0.05 |
|  | Bradycardias and conduction diseases | 1 | 10 | 0.57 |
|  | **COPD, emphysema and chronic bronchitis** | **13** | **40** | **2.24** |
|  | Cardiac valve diseases | 1 | 4 | 0.21 |
|  | Cataract and other lens diseases | 2 | 5 | 0.30 |
|  | Cerebrovascular diseases | 4 | 8 | 0.45 |
|  | Chronic kidney diseases | 27 | 12 | 0.69 |
|  | **Colitis and related diseases** | **26** | **38** | **2.14** |
|  | Deafness and hearing loss | 5 | 8 | 0.47 |
|  | **Depression and mood diseases** | **36** | **65** | **3.64** |
|  | Diabetes | 3 | 6 | 0.33 |
|  | **Dorsopathies** | **18** | **43** | **2.41** |
|  | Dyslipidemia | 47 | 16 | 0.88 |
|  | **Esophagus, stomach and duodenum diseases** | **11** | **37** | **2.05** |
|  | Glaucoma | 2 | 5 | 0.27 |
|  | Heart failure | <1 | <1 | 0.02 |
|  | Hypertension | 54 | 13 | 0.71 |
|  | Inflammatory arthropathies | 4 | 15 | 0.84 |
|  | Ischemic heart diseases | 4 | 4 | 0.25 |
|  | Migraine and facial pain syndromes | 4 | 28 | 1.56 |
|  | **Neurotic, stress-related and somatoform diseases** | **16** | **82** | **4.55** |
|  | Obesity | 9 | 12 | 0.65 |
|  | Osteoarthritis and other degenerative joint diseases | 22 | 27 | 1.49 |
|  | **Osteoporosis** | **17** | **40** | **2.24** |
|  | Other MSK joint diseases | 10 | 25 | 1.41 |
|  | Other cardiovascular diseases | 2 | 10 | 0.54 |
|  | Other eye diseases | 3 | 8 | 0.45 |
|  | Other genitourinary diseases | 1 | 9 | 0.50 |
|  | **Other neurological diseases** | **5** | **39** | **2.18** |
|  | Prostate diseases | 1 | 6 | 0.31 |
|  | **Sleep diseases** | **6** | **40** | **2.25** |
|  | Solid neoplasms | 7 | 12 | 0.67 |
|  | **Thyroid diseases** | **24** | **37** | **2.05** |

In bold are reported chronic conditions used to characterize and name the patterns (i.e., having O/E ratio of at least 2 and an exclusivity of at least 25%).

**Table S2.** Associations between multimorbidity patterns and blood biomarkers of Alzheimer’s disease considering participants without multimorbidity as the reference group

|  | **No multimorbidity (Ref.)** | **Unspecific** | **Psychiatric, Respiratory & MSK** | **Anemia & Sensory impairment** | **Cardiometabolic & Inflammatory** |
| --- | --- | --- | --- | --- | --- |
|  |  |  | β (95%CI) | β (95%CI) | β (95%CI) |
| **Aβ-42/40** | Ref. | -0.04 (-0.11; 0.03) | -0.01 (-0.09, 0.09) | -0.01 (-0.11, 0.08) | -0.10 (-0.21, 0.01) |
| **P-tau181** | Ref. | -0.00 (-0.08;0.07) | 0.02 (-0.08, 0.11) | **0.18 (0.08, 0.28)** | **0.24 (0.12, 0.36)** |
| **P-tau217** | Ref. | -0.02 (-0.07;0.04) | 0.01 (-0.06, 0.08) | **0.11 (0.03, 0.18)** | **0.23 (0.14, 0.32)** |
| **T-tau** | Ref. | -0.06 (-0.17;0.04) | -0.06 (-0.18, 0.07) | 0.07 (-0.07, 0.21) | **0.23 (0.07, 0.39)** |
| **NfL** | Ref. | **-**0.03 (-0.09;0.03) | 0.02 (-0.05, 0.09) | **0.14 (0.06, 0.21)** | **0.32 (0.23, 0.40)** |
| **GFAP** | Ref. | -0.02 (-0.06; 0.01) | 0.01 (-0.04, 0.04) | 0.03 (-0.01, 0.08) | 0.05 (-0.01, 0.10) |

β and 95%CIs are derived from quantile regression models on the 50^th^ (median) percentile, adjusted for age, sex, education.

Abbreviations: Aβ: amyloid beta; p-tau: phosphorylated tau; t-tau: total tau; NfL: neurofilament light chain; GFAP: glial fibrillary acidic protein; MSK: musculoskeletal.

**Table S3.** Concentration of blood biomarkers of Alzheimer’s disease (z-score) in relation to the number of chronic diseases at baseline after excluding those who developed dementia during the follow up

|  | **β (95%CI)** |
| --- | --- |
| **Aβ-42/40** | -0.012 (-0.015; 0.011) |
| **P-tau181** | **0.019 (0.007; 0.032)** |
| **P-tau217** | **0.011 (0.001; 0.021)** |
| **T-tau** | 0.005 (-0.013; 0.024) |
| **NfL** | **0.017 (0.009; 0.026)** |
| **GFAP** | 0.004 (-0.001; 0.012) |

β and 95%CIs are derived from quantile regression models on the 50^th^ (median) percentile, adjusted for age, sex, education.

Abbreviations: Aβ: amyloid beta; p-tau: phosphorylated tau; t-tau: total tau; NfL: neurofilament light chain; GFAP: glial fibrillary acidic protein.

**Table S4.** Associations between multimorbidity patterns and blood biomarkers of Alzheimer’s disease after excluding those who developed dementia during the follow-up

|  | **Unspecific (Ref.)** | **Psychiatric, Respiratory & MSK** | **Anemia & Sensory impairment** | **Cardiometabolic & Inflammatory** |
| --- | --- | --- | --- | --- |
|  |  | β (95%CI) | β (95%CI) | β (95%CI) |
| **Aβ-42/40** | Ref. | 0.03 (-0.06, 0.11) | -0.01 (-0.11, 0.08) | -0.06 (-0.18, 0.06) |
| **P-tau181** | Ref. | 0.02 (-0.06, 0.10) | **0.16 (0.07, 0.25)** | **0.20 (0.09, 0.31)** |
| **P-tau217** | Ref. | 0.02 (-0.04, 0.08) | **0.11 (0.04, 0.17)** | **0.27 (0.18, 0.35)** |
| **T-tau** | Ref. | 0.02 (-0.08, 0.13) | **0.13 (0.01, 0.25)** | **0.35 (0.19, 0.51)** |
| **NfL** | Ref. | **0.05 (0.00, 0.11)** | **0.12 (0.06, 0.19)** | **0.31 (0.23, 0.39)** |
| **GFAP** | Ref. | 0.03 (-0.04, 0.07) | **0.05 (0.04, 0.09)** | **0.09 (0.04, 0.15)** |

β and 95%CIs are derived from quantile regression models on the 50^th^ (median) percentile, adjusted for age, sex, education.

Abbreviations: Aβ: amyloid beta; p-tau: phosphorylated tau; t-tau: total tau; NfL: neurofilament light chain; GFAP: glial fibrillary acidic protein; MSK: musculoskeletal.

**Table S5.** Associations between multimorbidity patterns and blood biomarkers of Alzheimer’s disease considering the unspecific group as the reference

|  | **Unspecific (Ref.)** | **Psychiatric, Respiratory & MSK** | **Anemia & Sensory impairment** | **Cardiometabolic & Inflammatory** |
| --- | --- | --- | --- | --- |
|  |  | β (95%CI) | β (95%CI) | β (95%CI) |
| **Aβ-42/40** | Ref. | 0.03 (-0.04, 0.10) | 0.01 (-0.07, 0.08) | -0.07 (-0.17, 0.02) |
| **P-tau181** | Ref. | 0.02 (-0.06, 0.10) | **0.18 (0.09, 0.27)** | **0.24 (0.13, 0.34)** |
| **P-tau217** | Ref. | 0.03 (-0.04, 0.09) | **0.12 (0.05, 0.19)** | **0.23 (0.15, 0.32)** |
| **T-tau** | Ref. | 0.01 (-0.09, 0.11) | **0.14 (0.03, 0.24)** | **0.29 (0.16, 0.43)** |
| **NfL** | Ref. | 0.05 (-0.00, 0.11) | **0.16 (0.09, 0.22)** | **0.34 (0.25, 0.42)** |
| **GFAP** | Ref. | 0.02 (-0.02, 0.06) | **0.06 (0.02, 0.09)** | **0.07 (0.02, 0.12)** |

β and 95%CIs are derived from quantile regression models on the 50^th^ (median) percentile, adjusted for age, sex, education.

Abbreviations: Aβ: amyloid beta; p-tau: phosphorylated tau; t-tau: total tau; NfL: neurofilament light chain; GFAP: glial fibrillary acidic protein; MSK: musculoskeletal.

**Table S6**. Associations between multimorbidity patterns and blood biomarkers of Alzheimer’s disease, adjusted for kidney function.

|  | **Unspecific (Ref.)** | **Psychiatric, Respiratory & MSK** | **Anemia & Sensory impairment** | **Cardiometabolic & Inflammatory** |
| --- | --- | --- | --- | --- |
|  |  | β (95%CI) | β (95%CI) | β (95%CI) |
| **Aβ-42/40** | Ref. | 0.03 (-0.05, 0.10) | 0.01 (-0.06, 0.09) | -0.06 (-0.16, 0.04) |
| **P-tau181** | Ref. | 0.01 (-0.06, 0.08) | **0.14 (0.06, 0.21)** | **0.22 (0.11, 0.31)** |
| **P-tau217** | Ref. | 0.02 (-0.04;0.08) | **0.07 (0.01; 0.14)** | **0.21 (0.13; 0.30)** |
| **T-tau** | Ref. | 0.01 (-0.09, 0.12) | **0.13 (0.01, 0.24)** | **0.25 (0.10, 0.39)** |
| **NfL** | Ref. | 0.06 (-0.01, 0.12) | **0.15 (0.09, 0.22)** | **0.26 (0.18, 0.34)** |
| **GFAP** | Ref. | 0.02 (-0.02, 0.06) | **0.05 (0.00, 0.09)** | 0.04 (-0.02, 0.09) |

β and 95%CIs are derived from quantile regression models on the 50^th^ (median) percentile, adjusted for age, sex, education and glomerular filtration rate (eGFR).

Abbreviations: Aβ: amyloid beta; p-tau: phosphorylated tau; t-tau: total tau; NfL: neurofilament light chain; GFAP: glial fibrillary acidic protein¸ MSK: musculoskeletal.

**Table S7.** Associations between multimorbidity patterns and blood biomarkers of Alzheimer’s disease, stratified by age.

|  |  | **Unspecific (Ref.)** | **Psychiatric, Respiratory & MSK** | **Anemia & Sensory impairment** | **Cardiometabolic & Inflammatory** |
| --- | --- | --- | --- | --- | --- |
|  |  |  | β (95%CI) | β (95%CI) | β (95%CI) |
| **Aβ-42/40** | *<75 years old* | Ref. | 0.09 (-0.01, 0.18) | 0.03 (-0.01, 0.18) | -0.02 (-0.23, 0.19) |
|  | *≥75 years old* | Ref. | -0.04 (-0.15, 0.08) | **-**0.03 (-0.20, 0.00) | -0.10 (-0.20, 0.00) |
| **P-tau181** | *<75 years old* | Ref. | -0.00 (-0.07, 0.07) | **0.16 (0.06, 0.25)** | **0.46 (0.32, 0.61)** |
|  | *≥75 years old* | Ref. | 0.15 (-0.03, 0.32) | **0.24 (0.10, 0.38)** | **0.19 (0.03, 0.34)** |
| **P-tau217** | *<75 years old* | Ref. | 0.00 (-0-04;0.05) | 0.04 (-0.02;0.10) | **0.26 (0.17;0.36)** |
|  | *≥75 years old* | Ref. | 0.05 (-0.12;0.22) | **0.20 (0.07;0.33)** | **0.23 (0.08;0.39)** |
| **T-tau** | *<75 years old* | Ref. | 0.03 (-0.09, 0.15) | 0.10 (-0.06, 0.27) | **0.38 (0.13, 0.63)** |
|  | *≥75 years old* | Ref. | -0.05 (-0.25, 0.16) | 0.14 (-0.02, 0.30) | **0.27 (0.09, 0.46)** |
| **NfL** | *<75 years old* | Ref. | **0.05 (0.01, 0.08)** | **0.11 (0.06, 0.17)** | **0.11 (0.03, 0.20)** |
|  | *≥75 years old* | Ref. | 0.04 (-0.11, 0.19) | **0.12 (0.01, 0.24)** | **0.25 (0.12, 0.38)** |
| **GFAP** | *<75 years old* | Ref. | 0.02 (-0.01, 0.05) | 0.03 (-0.02, 0.07) | **0.08 (0.02, 0.15)** |
|  | *≥75 years old* | Ref. | 0.03 (-0.07, 0.13) | 0.06 (-0.02, 0.14) | 0.04 (-0.05, 0.14) |

β and 95%Cis are derived from quantile regression models on the 50^th^ (median) percentile, adjusted for sex and education.

Abbreviations: Aβ: amyloid beta; p-tau: phosphorylated tau; t-tau: total tau; NfL: neurofilament light chain; GFAP: glial fibrillary acidic protein¸ MSK: musculoskeletal.

**Table S8.** Associations between multimorbidity patterns and blood biomarkers of Alzheimer’s disease, stratified by sex.

|  |  | **Unspecific (Ref.)** | **Psychiatric, Respiratory & MSK** | **Anemia & Sensory impairment** | **Cardiometabolic & Inflammatory** |
| --- | --- | --- | --- | --- | --- |
|  |  |  | β (95%CI) | β (95%CI) | β (95%CI) |
| **Aβ-42/40** | *Males* | Ref. | 0.10 (-0.05, 0.26) | 0.00 (-0.14, 0.14) | -0.11 (-0.28, 0.06) |
|  | *Females* | Ref. | 0.03 (-0.05, 0.11) | 0.04 (-0.06, 0.13) | -0.04 (-0.16, 0.08) |
| **P-tau181** | *Males* | Ref. | -0.02 (-0.17, 0.13) | **0.27 (0.13, 0.41)** | **0.42 (0.26, 0.59)** |
|  | *Females* | Ref. | 0.03 (-0.06, 0.12) | **0.12 (0.02, 0.22)** | 0.08 (-0.04, 0.21) |
| **P-tau 217** | *Males* | Ref. | -0.01 (-0.14;0.10) | **0.18 (0.07;0.30)** | **0.34 (0.21;0.48)** |
|  | *Females* | Ref. | 0.02 (-0.05;0.10) | 0.08 (-0.01;0.16) | **0.19 (0.07;0.30)** |
| **T-tau** | *Males* | Ref. | 0.01 (-0.17, 0.19) | **0.24 (0.08, 0.41)** | **0.50 (0.31, 0.70)** |
|  | *Females* | Ref. | 0.00 (-0.14, 0.13) | 0.08 (-0.06, 0.23) | 0.16 (-0.03, 0.35) |
| **NfL** | *Males* | Ref. | 0.05 (-0.06, 0.16) | **0.22 (0.12, 0.32)** | **0.39 (0.27, 0.51)** |
|  | *Females* | Ref. | 0.04 (-0.03, 0.12) | **0.11 (0.03, 0.19)** | **0.30 (0.19, 0.40)** |
| **GFAP** | *Males* | Ref. | 0.02 (-0.05, 0.08) | **0.09 (0.03, 0.15)** | **0.11 (0.04, 0.18)** |
|  | *Females* | Ref. | 0.03 (-0.02, 0.09) | 0.03 (-0.03, 0.09) | 0.03 (-0.04, 0.10) |

β and 95%Cis are derived from quantile regression models on the 50^th^ (median) percentile, adjusted for age and education.

Abbreviations: Aβ: amyloid beta; p-tau: phosphorylated tau; t-tau: total tau; NfL: neurofilament light chain; GFAP: glial fibrillary acidic protein¸ MSK: musculoskeletal.

**Figure S1**. Flow chart of the study.


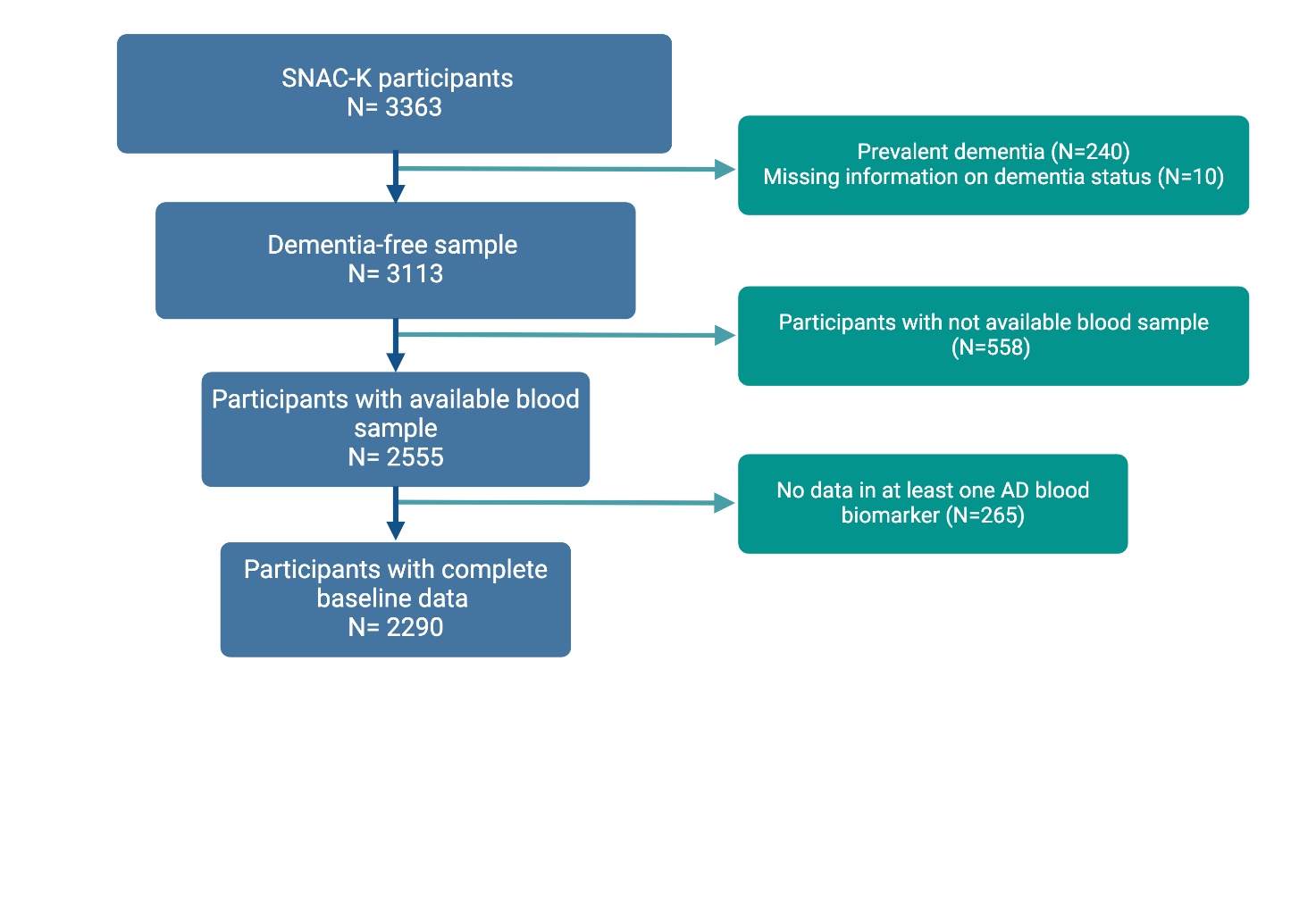

Supplement: Supplementary file 1 — Supporting information [file ALZ-21-e70411-s002.docx]
